# Assessment of immune responses to a Comirnaty^®^ booster following CoronaVac^®^ vaccination in healthcare workers

**DOI:** 10.1590/0074-02760230239

**Published:** 2024-09-09

**Authors:** Lucas Bochnia-Bueno, Gabriela Mattoso Coelho, Allan Henrique Depieri Cataneo, Camila Zanluca, Laura Holtman Ferreira, Luciano Pamplona de Goes Cavalcanti, Marco Antonio de Freitas Clementino, Juliana Navarro Ueda Yaochite, Hellen Geremias dos Santos, Meri Bordignon Nogueira, Claudia Nunes Duarte dos Santos, Sonia Mara Raboni

**Affiliations:** 1 Universidade Federal do Paraná, Laboratório de Virologia, Curitiba, PR, Brasil; 2 Universidade Federal do Paraná, Programa de Pós-Graduação em Microbiologia, Parasitologia e Patologia, Curitiba, PR, Brasil; 3 Fundação Oswaldo Cruz-Fiocruz, Instituto Carlos Chagas, Laboratório de Virologia Molecular, Curitiba, PR, Brasil; 4 Universidade Federal do Ceará, Faculdade de Medicina, Fortaleza, CE, Brasil; 5 Universidade Federal do Ceará, Faculdade de Farmácia, Odontologia e Enfermagem, Departamento de Análises Clínicas e Toxicologia, Fortaleza, CE, Brasil; 6 Fundação Oswaldo Cruz-Fiocruz, Instituto Carlos Chagas, Curitiba, PR, Brasil

**Keywords:** SARS-CoV-2, vaccine, immune response, immunization, public health, pandemic

## Abstract

**BACKGROUND:**

The immunological response to severe acute respiratory syndrome coronavirus 2 (SARS-CoV-2) infection and immunisation is variable.

**OBJECTIVES:**

To describe the humoral immune response by correlating IgA and IgG antibodies with NAbs titration following CoronaVac^®^ immunisation and an mRNA (Comirnaty^®^) booster among healthcare workers (HCWs) and to compare the cytokine and interleukin profiles between HCWs vaccinated with CoronaVac and coronavirus disease 2019 (COVID-19) infected patients.

**METHODS:**

Samples from 133 HCWs collected at 20 (T1) and 90 (T2) days after CoronaVac immunisation and 15 (T3) days after a booster dose with the Comirnaty vaccine were analysed for IgA and IgG EIA and neutralisation assay. Cytokine levels from vaccinated individuals at T1 day and COVID-19 patients were compared.

**FINDINGS:**

Neutralising antibodies (NAbs) were observed in 81.7% of participants at T1, but only 49.2% maintained detectable NAbs after 90 days. The booster dose increased NAbs response in all participants. The cytokines with the highest levels post-vaccination were IL-6 and MCP-1. The MCP-1, IL-18, and IFN- γ levels were higher in COVID-19 patients than in vaccinated HCWs, while IL-22 levels increased in the vaccinated HCWs group.

**MAIN CONCLUSIONS:**

The neutralisation titres in the T2 samples decreased, and antibody levels detected at T2 showed a more significant reduction than the neutralisation. The higher IL-22 expression in immunised individuals compared to those with COVID-19 suggests that IL-22 may be beneficial in protecting against severe disease.

Three years into the severe acute respiratory syndrome coronavirus 2 (SARS-CoV-2) pandemic, the global count has surpassed 763 million confirmed cases, with over 6.9 million deaths.^1^ The emergence of this novel coronavirus in December 2019 triggered a global search for an effective vaccine to combat the coronavirus disease 2019 (COVID-19) pandemic, resulting in a range of vaccines based on diverse antigen platforms.^2^ ^,^ ^3^

During clinical trials, vaccines demonstrated distinct efficacy, with mRNA and non-replicating viral vectors outperforming inactivated virus products.^2^ ^,^ ^3^ Due to the widespread use of these vaccines in high-income countries (HIC), low- and middle-income countries (LMIC) primarily accessed inactivated viral vaccines. The CoronaVac^®^ (Sinovac Life Sciences, Beijing, China), which uses the inactivated SARS-CoV-2 virus^2^ ^,^ ^3^ represented 22.15% of the total doses administered in Brazil until April 24th, 2023, being the second most administered vaccine among healthcare workers (HCWs).^4^ Phase I/II studies indicated this vaccine was safe, tolerable, had adequate immunogenicity, and rare adverse reactions, and 97% of participants aged 18-59 years old had seroconversion.^5^ In phase III trials among healthcare workers, this vaccine showed 50.7%, 83.7%, and 100% efficacy against symptomatic illness, cases requiring medical assistance, and severe cases, respectively.^6^ ^,^ ^7^

The Comirnaty^®^ vaccine, also named BNT162b2 (BioNTech and Pfizer), consists of a nucleoside-modified mRNA encoding the viral spike glycoprotein of SARS-CoV-2, encapsulated in lipid nanoparticles. Administration of Comirnaty^®^ in Brazil began in May 2021.^4^ Phase I/II/III clinical trial results showed 94.6% protective efficacy in patients aged 16-85.^8^

According to previous studies on vaccination strategies, the same antigen platform systems, known as homologous immunisation, are often utilised for booster vaccination. In contrast, some researchers have presented sequential immunisation strategies for heterologous initial and booster vaccination against COVID-19.^9^ ^,^ ^10^ ^,^ ^11^ Studies have suggested that mixed vaccination schedules for COVID-19 vaccines may lead to higher antibody levels and a more comprehensive immune response, outperforming standard vaccination schedules without causing severe side effects when compared to those caused by the vaccine’s default schemes.^12^ ^,^ ^13^

Serum antibody prevalence, including IgG and IgA, is crucial for monitoring infection and vaccination coverage.^14^ ^,^ ^15^ However, not all antibodies induced by immunisation have a neutralising capacity. Thus, quantifying neutralising antibodies (NAbs) is the most appropriate approach to ensure adequate humoral protection against infection.^16^ Despite these benefits, the turnaround time, cost, and biosafety risks make them unsuitable for serological surveillance.

Immunity against SARS-CoV-2 may be induced by several pathways, such as vaccine-induced and natural immunity following infection. Although both provide some level of immunisation, there is still no consensus in the literature on which confers more effective protection.^17^

Given the heterogeneity of the immune response in SARS-CoV-2 infection and vaccination, it is necessary to understand the relationship between the inflammatory response and post-vaccination antibody response. This study aimed to describe the humoral immune response by correlating IgA and IgG antibodies with NAbs titres following CoronaVac^®^ immunisation and an mRNA (Comirnaty^®^) booster among HCWs, and to describe the cytokines and interleukins profile antibodies by comparing the response between HCWs CoronaVac vaccinated and COVID-19 infected patients.

## MATERIALS AND METHODS

*Participants* - In total, 170 participants were recruited at the Complexo Hospital de Clínicas, Universidade Federal do Paraná (CHC/UFPR), in Curitiba, Brazil, during the HCWs vaccination period. The Institutional Research Ethics Board approved the study, and the participants signed the informed consent (# 31687620.2.0000.0096).

The inclusion criteria were answering a questionnaire, receiving two doses of CoronaVac^®^ (at intervals ranging from 15 to 21 days - Primary immunisation), receiving a booster dose of the Comirnarty^®^ vaccine (six to eight months after the first dose of vaccine), and providing serum samples. Fourteen participants were excluded for not completing the questionnaire. Additionally, seven participants received another vaccine, one did not have the second dose, and 15 did not provide a sample on day 20 post-vaccination.

Serial serum samples of 133 HCWs included in this study were collected on days 20 (T1) and 90 (T2) after the second dose and 15 (T3) days after the Comirnaty^®^ booster dose. The time intervals were defined to analyse the immune response at its peak, which occurs around two to three weeks after the proposed immunisation is completed. T1 had 133 serum samples, T2 and T3 included 132 and 117 serum samples, respectively. One and 16 patients were dropouts at T2 and T3, respectively, as they did not provide blood samples for evaluation. All samples were stored at -20ºC until analysis was performed.

*Evaluation of antibody seroconversion* - Serum samples were analysed in duplicate using a quantitative enzymatic immunoassay (EIA) for IgG and semi-quantitative assays for IgA anti-S1 spike protein receptor-binding domain (RBD) (Euroimmun, Lübeck, Germany), catalogue number EI2606 G (IgG) and EI2606 A (IgA), following the manufacturer’s instructions. Duplicates with a variation coefficient greater than 15% for absorbance were repeated. The immunoglobulins quantitative results of this cohort have been previously reported.^18^ ^,^ ^19^ ^,^ ^20^ Here, these results were compared with cytokine quantification and the neutralisation titres.

*Fluorescence reduction neutralisation assay (FRNA)* - Serum samples were serially diluted from 1:20 to 1:10,240 in DMEM F-12 medium with 100 IU/mL penicillin and 100 µg/mL streptomycin, except for foetal bovine serum. Each dilution was mixed with 450 PFU of SARS-CoV-2 (GenBank: MT807936.1) stock and incubated at 37ºC for 1 h. The mixtures were added to 96-well plates containing Vero E6 cells (5 x 10^4^ cells/well) and were incubated at 37ºC and 5% CO2 for 24 h in DMEM F-12 supplemented with 10% foetal bovine serum and antibiotics. Afterward, cells were fixed in methanol/acetone (1:1 v/v) for at least 1 h at -20ºC. Immunofluorescence assay (IFA) was performed using an in-house anti-SARS-CoV-2 monoclonal antibody, followed by anti-mouse IgG Alexa Fluor 488 conjugated antibody (Thermo Fisher Scientific, Grand Island, USA) and DAPI (4,6-diamidino-2-phenylindole; Invitrogen, Waltham, MA, USA).^20^ IFA images were captured using an automated imaging system (Operetta CLS High-Content Imaging System; Perkin Elmer) with a 20x objective. The images were analysed using the Harmony High-Content Imaging and Analysis Software (Perkin Elmer) to determine the proportion of infected cells. The assay quality was assessed by calculating the Z factor (Z’ = 1- [3(σp+σn)/(μp- μn)]; σ is the standard deviation, and μ is the mean of positive (p) and negative (n) controls). Results with Z ≥ 0.5 were used to calculate neutralising titres. Infection was normalised using positive and mock controls, and curves were generated using the log(inhibitor) vs. normalised response variable slope model in Prism software (GraphPad) to calculate the serum dilution that inhibited 50% of SARS-CoV-2 infection (NT_50_). Samples with NT_50_ higher than 20 were considered positive.

*Cytokines quantification* - HCWs’ cytokine levels were measured 20 days following the second vaccine dose (T1). To compare cytokine production between immunisation and SARS-CoV-2 infection, we conducted a study with 120 patients admitted to the CHC-UFPR with moderate to severe COVID-19. Blood samples were obtained within 10 ± 2 days of the onset of symptoms, and these patients were hospitalised between June/2020 and December/2020, before the Brazilian vaccination campaign.

Quantification of serum cytokines GM-CSF, MCP-1, TNF-α, IFN-α, IFN-γ, IL-1β, IL-10, IL-13, IL-17A, IL-18, IL-2, IL-21, IL-22, IL-23, IL-27, IL-4, IL-5, IL-6 and IL-9 was performed using the custom kit Human ProcartaPlex Multiplex Immunoassay (Thermo Fisher Scientific, MA, USA). The measurement was performed using the MAGPIX System (Merck, MA, USA) based on Luminex xMAP technology, according to the manufacturer’s instructions.

A heatmap was developed using the GraphPad Prism software, comparing the median of cytokine concentration (pg/mL) from COVID-19 patients and vaccinated HCWs.

Statistical analysis

*Graph profile and mixed linear model* - The neutralisation values for T1, T2, and T3 were presented in a graph profile. A mixed linear model was adjusted to estimate each period’s mean and respective 95% confidence interval (95%CI). The linear mixed model incorporates both a random term to represent repeated measures in the same individual and fixed parameters, corresponding to the mean values of the variable at each level of the qualitative variable.^21^ Due to the substantial positive asymmetry of the neutralisation data and values observed equal to zero for T1, we applied the log(x+1) transformation before adjusting the mixed linear model. Ratio comparisons between the mean of the three times were estimated with the corresponding 95%CI.

*Bivariate analysis* - For each time, the Spearman correlation coefficient and its corresponding 95%CI were used to examine the relationship between neutralisation values and IgG and IgA. Additionally, the distribution of the neutralisation values between sex (male and female), risk factors (yes or no), and age groups (less than 50 and 50 years or more) were assessed. The Wilcoxon test was used to summarise the differences between categories of each variable. Those with a p-value < 5% were considered statistically significant.

*Receiver operating characteristic curve (ROC curve) analysis* - The FRNA data were dichotomised into positive (titration ≥ 20) or negative (titration < 20) and used as the gold standard for IgG and IgA immune response. The distribution of each immunoglobulin was examined according to neutralisation categories (positive or negative), and a logistic regression model was adjusted separately for each one, aiming to determine a corresponding cut-off point that can identify the level of antibodies required for immune protection (Protection correlate). Four models were adjusted, two for IgG (T1 and T2) and two for IgA (T1 and T2). T3 time was not adjusted for this analysis as all participants were classified as positive (presented neutralisation values above 20). A combined logistic regression and leave-one-out cross-validation (CV) process was applied to adjust each model. The observations were randomly divided into training and test data on each CV iteration. The former was used to adjust a logistic regression model, and the latter to estimate the probability of being classified as a positive sample. The training and test data included all samples, and the vector of estimated probabilities was used to evaluate model performance by selecting a corresponding cut-off point that maximised the model’s sensitivity and specificity. The IgG and IgA values for each time (T1 and T2) corresponding to the estimated probability cut-off point were calculated. Each sample was then classified as positive or negative according to the chosen cut-off. The following measures and their corresponding 95%CI were calculated: AUROC curve, Sensitivity, Specificity, Positive Predictive Value, and Negative Predictive Value. The IgG and IgA index were examined individually in GraphPad Prism against the results of each cytokine to obtain a simple linear regression (Spearman Correlation).

## RESULTS

**Fig. 1:**
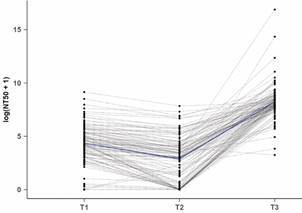
distribution of neutralisation titres that inhibit 50% of severe acute respiratory syndrome coronavirus 2 (SARS-CoV-2) infection (NT_50_). Paired samples from 114 serum samples were collected at three different collection times. Note: T1: 20 days post-primary. We adjusted a linear mixed model to estimate the mean NT_50_ for each data point and compared the obtained values between points.

*Comparison between FRNA and IgG and IgA results* - Fig. 1 shows the mean profile of FRNA results between T1 and T3. Most participants (82%) developed neutralising antibodies two weeks after completing their initial immunisation schedule (T1). On day 90 after vaccination (T2), these titres decreased significantly between complete immunisation and booster dose [Supplementary data (Tables I-II)]. Compared to T1, the mean of NT_50_ at T2 decreased from 81.7 (95%CI: 54.1; 123.2) to 18.2 (95%CI: 11.8; 27.8) [Supplementary data (Table I)]. Furthermore, only 49.2% of participants had detectable titres, a 32.8% decrease. Booster vaccination increased the percentage of participants with detectable neutralisation from 49.2% to 97.4% (including five participants who had non-neutralising antibodies before) and increased NAb titres, with an estimated NT_50_ mean at T3 of 3,831.1 (95%CI: 2,550.5;5754.4).

It was observed that the neutralisation titres and the concentration of IgG and IgA antibodies showed a moderate direct correlation when comparing the results obtained from the collections at different times following vaccination [Supplementary data (Fig. 1)]. However, IgG antibodies exhibited a stronger correlation with neutralisation (T1: rho = 0.60, 95%CI: 0.42;0.74; T2: rho: 0.64, 95%CI: 0.47; 0.78 and T3: rho = 0.60, 95%CI: 0.40; 0.76) compared to IgA antibodies (T1: rho = 0.44, 95%CI: 0.22; 0.63; T2: rho = 0.41, 95%CI: 0.16; 0.60 and T3: rho = 0.48, 95%CI: 0.27; 0.65) [Supplementary data (Fig. 1, Table III)].

**Fig. 2:**
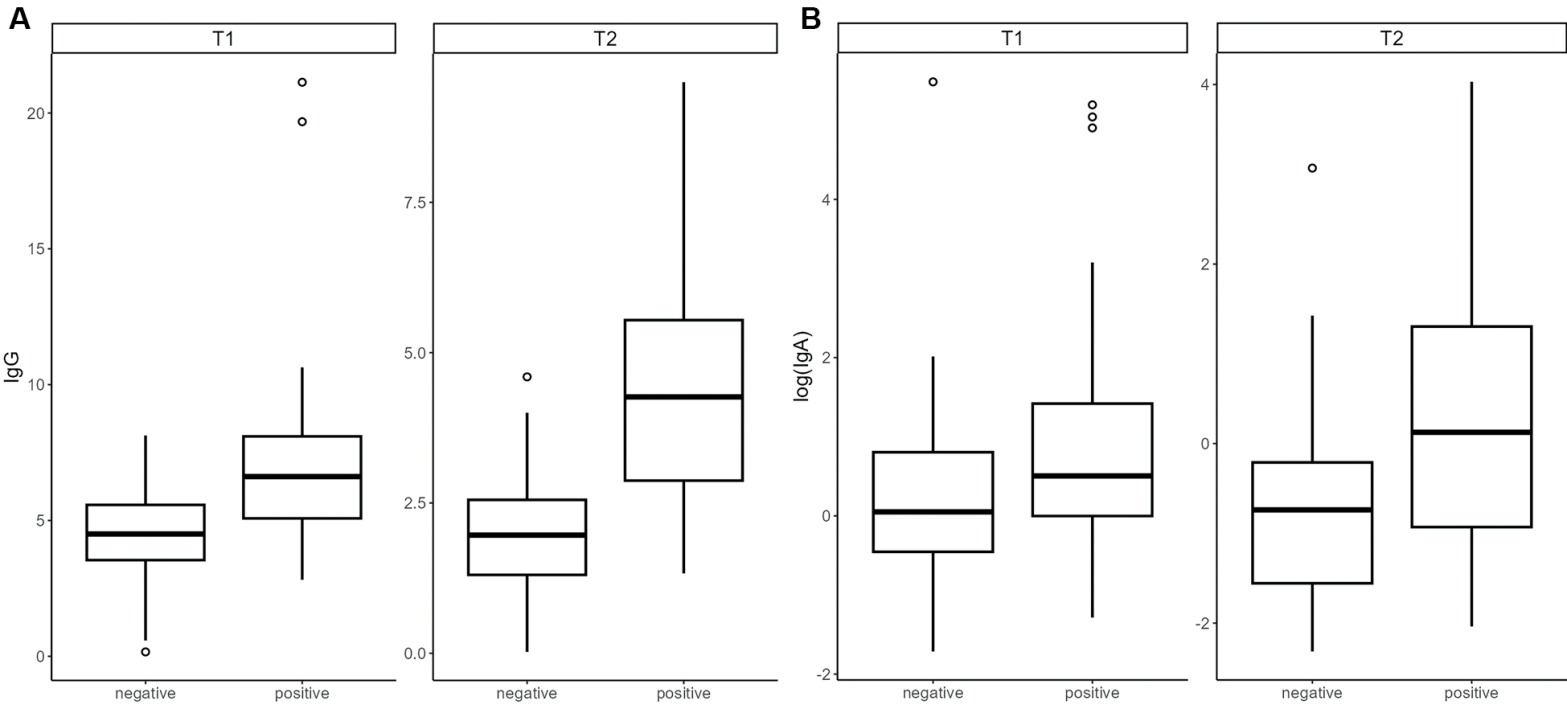
comparison between the titres of IgG (A) and IgA (B) anti-severe acute respiratory syndrome coronavirus 2 (SARS-CoV-2) protein S antibodies in sera with negative (NT_50_ < 20) and positive (NT_50_ > 20) neutralisation titres at two different collection times. Note: T1: 20 days post-primary vaccination; T2: 90 days post-primary vaccination; NT_50_: neutralisation titres that inhibit 50% of SARS-CoV-2 infection.

**Fig. 3:**
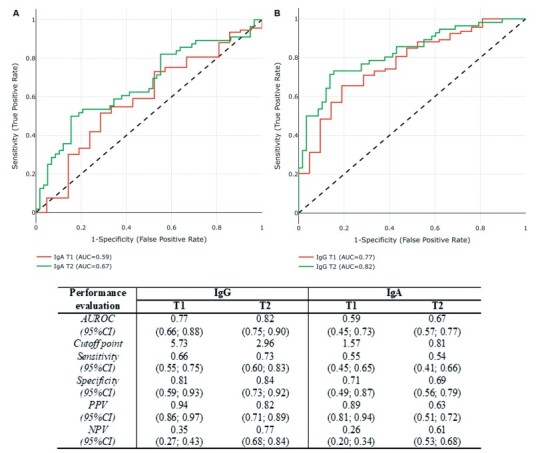
receiver operating characteristic (ROC) curve of the IgA (A) and IgG (B) anti- severe acute respiratory syndrome coronavirus 2 (SARS-CoV-2) S1 protein antibodies in two different collection times. The table shows the results obtained from the performance of the adjusted models. Note: T1: 20 days post-primary vaccination; T2: 90 days post-primary vaccination; AUC: area under the ROC curve.

Figure 2 shows the distribution of IgG (A) and IgA (B) antibody levels between samples positive and negative for neutralisation (*i.e.*, neutralisation-positive samples had overall higher levels of IgG than negative ones) at T1 and T2. Considering NT_50_ greater than or equal to 20 as positive, an attempt was made to determine a cut-off value for IgG and IgA concentrations that could be considered as correlates of protection (Fig. 3). ROC curves were estimated and IgG cut-off values of 5.73 and 2.96 were obtained at T1 and T2, with AUCs of 0.77 and 0.82, respectively. For IgA, cut-off values of 1.57 and 0.81 were found at T1 and T2, with AUCs of 0.57 and 0.67, respectively (Fig. 3). Additionally, we compared the NT_50_ titres according to sex, presence of risk factors, and age. No differences were found [Supplementary data (Fig. 2, Table IV)].

**Fig. 4:**
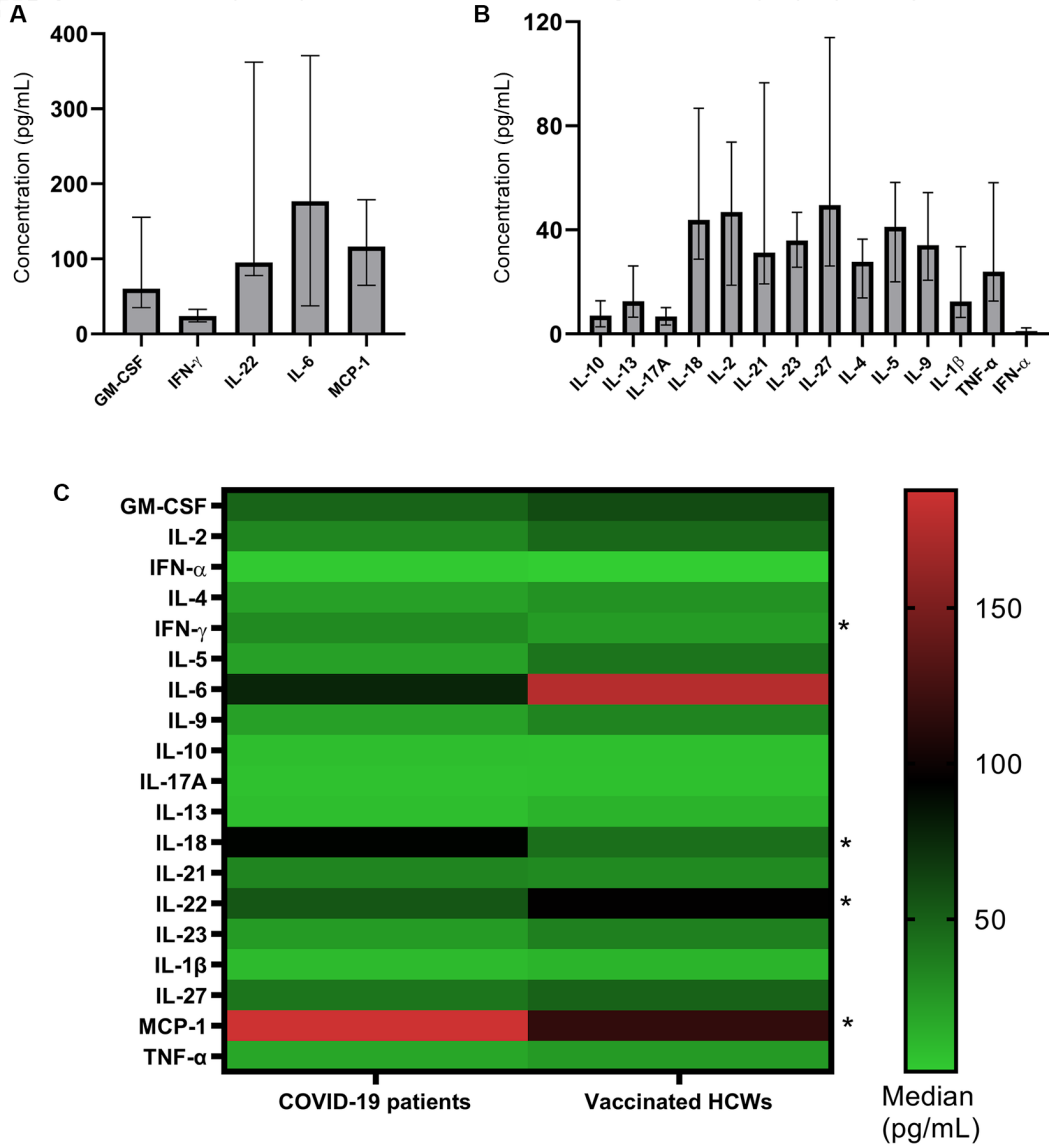
cytokine profile of healthcare workers (HCWs) on the 20th day post-vaccination (T1). A) Altered cytokines at high concentrations. B) Altered cytokines in lower concentrations. The detectable values are shown as median and interquartile ranges in different scales. C) Heatmap of cytokine levels (Median, pg/mL) in coronavirus disease 2019 (COVID-19) patients and vaccinated HCWs (*p < 0,05; Mann Whitney test). Note: MCP-1: monocyte chemoattractant protein-1; IL: interleukin; IFN: interferon; GM-CSF: granulocyte-macrophage colony-stimulating factor; TNF: tumour necrosis factor.

*Cytokines production and correlation with IgG and IgA concentrations* - HCWs vaccinated against SARS-CoV-2 were assessed for producing 19 cytokines. Among them, IL-6 and MCP-1 presented the highest levels 20 days after the second immunisation (T1), with medians of 176.7pg/mL (37.59-370.9) and 116.6pg/mL (64.73-178.7), respectively (Fig. 4A-B). The production of cytokines was not evaluated in T3 samples.

To evaluate the cytokines production during “artificial infection” (immunisation) and “natural infection” (viral infection), the levels of these mediators were compared between vaccinated HCWs and COVID-19 patients (around 10 days’ worth of symptoms) [Supplementary data (Table V)]. The mediators MCP1, IFN-γ, and IL-18 stand out among those in which increased production was observed in COVID-19 patients. On the contrary, IL-22 levels were increased in the vaccinated HCWs group (Fig. 4C).

We assessed the MCP-1, IFN-γ, IL-10, and IL-18 levels concerning the IgA and IgG responses against SARS-CoV-2 as a comparative analysis. The results showed no correlation [Supplementary data (Figs 3-4).

## DISCUSSION

This study aimed to investigate the humoral immune response among HCWs in Brazil who received the CoronaVac^®^ vaccine followed by a booster dose of the Comirnaty^®^ vaccine. To assess the extent and durability of the response to the initial vaccination and the effect of the booster dose, we measured titres of NAbs and IgG and IgA antibodies approximately 20 and 90 days after the second CoronaVac dose, and 15 days after the Comirnaty dose. Moreover, we examined correlations between NAb titres and IgG or IgA antibody levels and built ROC curves to investigate whether IgG or IgA cut-off values could be used as correlates of neutralisation. Finally, the cytokine profile of the vaccinated participants was also analysed and compared with those of hospitalised COVID-19 patients.

Overall, the seroconversion rate among the studied group was very satisfactory, with 81.9% of the subjects presenting neutralising antibodies after completing the initial immunisation scheme. Such response was not influenced by age, sex, and the presence of risk factors in the studied group. Approximately three months after the initial vaccination, a significant reduction in these titres was observed and half of the participants exhibited undetectable neutralising titres. Interestingly, a heterologous booster dose led to much higher titres than the initial immunisation, even leading to seroconversion of the previously unresponsive subjects. This finding was similar to the responses observed in individuals who were primarily immunised with mRNA vaccines.^22^ When anti-S IgG and IgA antibody titres were compared to neutralising antibody titres, a moderate positive correlation was observed mainly with IgG. ROC curves showed cut-off values with a sensitivity and specificity close to 70% and 80%, respectively, which might be used as a correlate of protection. These findings show that anti-S IgG titres provide protection against infections for three to four months post-immunisation and can be used to identify individuals who require new doses of immunisers.

The decrease in NAbs titres observed 110 days after the first dose is consistent with previous studies indicating that neutralising antibodies anti-SARS-CoV-2 may decline over time after vaccination.^23^ ^,^ ^24^ Previous studies suggested a booster dose for individuals vaccinated with CoronaVac due to the vaccine’s lower NAbs titre.^25^ Our study found that a boost dose increased the NAbs response with detection in nearly all participants.

NAbs play a crucial role in protecting against SARS-CoV-2 infection by preventing the virus from infecting host cells. NAbs have been associated with reduced risk of reinfection and severe COVID-19 outcomes.^26^ Nab detection assays typically rely on wild-type viruses, which demand BSL-3 laboratories. Alternatively, pseudoviruses or viral vectors expressing viral proteins may be used; however, they may not fully represent the complexity of the pathogen.^27^ Therefore, it is essential to identify alternative correlates of protection. Measurement of specific IgG and IgA antibodies against SARS-CoV-2 may be a suitable parameter to protection markers. However, further research is imperative to establish a minimum threshold of immunoglobulins associated with effective protection against SARS-CoV-2. This involves studying a larger and diverse population, conducting clinical and laboratory monitoring of participants, thereby validating the estimates proposed in this study. Additionally, exploring various populations and cultures is crucial for a comprehensive understanding.

Patients with SARS-CoV-2 infection present SARS with diffuse alveoli damage. The injury pattern of the disease is a consequence of an intense inflammatory response activating the complement system and innate immune cells, resulting in a massive cytokine release. The immune profile of patients with COVID-19 has been widely studied, and both dysfunction in innate and adaptive immunity have been observed.^28^ ^,^ ^29^ CD16+ monocytes, γδ T cells, and NK cells are activated, but CD4+ T cells, CD8+ T cells, and natural killer (NK) cells are considerably decreased.^29^ Furthermore, T cells exhibit hyperinflammatory responses, enhanced migratory abilities, and markedly increased expression of inhibitory molecules. B cell clonality is also high, as is the proportion of plasma B cell compartments. Dendritic cell percentages are decreased. However, cellular compartments responsive to IFN increased. Several studies have shown that the IL-1 and IL-6 axes are the most relevant signal transductions in SARS-CoV-2-induced hyperinflammatory responses.^28^ ^,^ ^30^

Currently, knowledge regarding the immunity generated by COVID-19 vaccines is insufficient, limiting the development of new generations of vaccines. Relevant concerns have been raised regarding the potential of immunisations to provide long-term protection, and whether individuals who have recovered from COVID-19 infection acquire immune defences comparable to or additional to vaccine-induced immunity.^31^

After COVID-19 infections, natural immunity offers some protection against reinfection, albeit it has been established that this protection is less effective against Omicron. Immunising individuals who have recovered from COVID-19 has the added benefit of strengthening their protective immunity against variants like Omicron.^31^

In this study, we compared a broad profile of cytokines in blood samples from vaccinated subjects to a case series of hospitalised COVID-19 patients. Sera were collected from patients who had symptoms for approximately ten days, and samples were collected at T2 of the group under study (HCWs), corresponding to about 20 days following initial immunisation. IL-6 and MCP-1 were found at higher levels in both groups, whereas MCP-1, IFN-g and IL-18, stood out among those with increased production in COVID-19 patients, compared to the vaccinated individuals. However, IL-22 levels increased significantly among vaccinated healthcare workers.

Pinto et al.^32^ reported increased levels of IL-6, IL-10, and CCL2/MCP-1 in the COVID-19 patients who died. Additionally, pro-inflammatory and antiviral mediators were imbalanced in the studied groups, with IL-6 and CCL2/MCP-1 strongly associated with the illness.^32^ According to Lee et al.^33^ the levels of nine cytokines (CCL2/MCP-1, CCL4, CXCL10, GM-CSF, IL-10, IL-17A, IL-6, IL-8, and TNF) were significantly higher in patients who died compared to COVID-19 survivors.^33^ Yaochite and colleagues^34^ evaluated the production of twenty cytokines upon hospital admission. In comparison to those who recovered, the fatal cases had considerably higher levels of IL-18, indicating that this mediator may be a possible marker for predicting poor prognosis in critically ill patients with COVID-19.^35^

Numerous studies have described the serum proteome of COVID-19 patients, frequently stratified by disease severity and comorbidities, showing substantial changes in immune-inflammatory pathways. However, results vary across studies, and the potential proteins in the so-called cytokine storm syndrome vary significantly depending on the community´s demographics. Suhre and colleagues examined changes in protein expression in COVID-19 patients across five case-control studies. Comparative proteome analysis was used by the authors to identify numerous inflammatory cytokines that were statistically overexpressed and might be targeted to prevent adverse immune-inflammatory effects in COVID-19 patients. These inflammatory cytokines included IL-6, IL-18, CCL7, CXCL10, and CXCL11.^36^ Following prime-boost vaccination, Covaxin/BBV152 (whole virion inactivated SARS-CoV-2 vaccine) immunisation elicits increased cytokine (IFN-γ, IL-2, TNFα, L-6, IL-12, IL-1α, IL-1β, IL-3, and IL-7) and chemokine (CCL4, CXCL1, CXCL2, and CX3CL1) release as early as month 1, demonstrating effective activation of innate and adaptive immune responses in immunised recipients.^37^

Wang and colleagues performed a scRNA-seq in PBMCs from CoronaVac-immunised individuals and compared the results to the single-cell profiles in COVID‐19 patients. The authors found that CoronaVac immunisation caused a weaker inflammatory response than natural SARS-CoV-2 infection since the expression of pro-inflammatory cytokine genes was significantly lower in the artificial setting.^38^

The weak immune response observed in inactivated-virus vaccines compared to wild viruses may be explained by the limited duration of the presented antigens and the lack of dead infected cell fragments for MHC I presentation of the viral antigens.^39^

In this study, the vaccination promoted an increased IL-22 production compared to that observed in COVID-19 patients. IL-22, a member of the IL-10 family of cytokines, has standard receptor features and has attracted research interest in recent years. Cells of the lymphoid lineage are the leading producers of IL-22, encompassing innate and adaptive immune system cells.^28^ ^,^ ^40^ Unlike most cytokines, which target haematopoietic cells, IL-22 acts primarily on non-haematopoietic epithelial cells and fibroblasts in a variety of tissues, inducing proliferation, inhibiting apoptosis of epithelial cells, and leading to the production of antimicrobial molecules. IL-22 can also induce the expression of pro-inflammatory molecules, including IL-1, IL-6, IL-8, IL-11, GCSF, GM-CSF, and LPS binding protein.^41^ ^,^ ^42^ As a result, while this interleukin may have a protective function in tissues, it may also be pro-inflammatory. Whether IL-22 will have a protective or pro-inflammatory effect depends on the related cytokines co-produced by the relevant cells during different disease stages.^43^ ^,^ ^44^

Regarding IL-22 expression in COVID-19 patients, Albayrak et al. have shown that SARS-CoV-2 infection is characterised by an abnormal expression of IL-22R1 in blood myeloid cells and CD4+ T lymphocytes, suggesting that the IL-22R1/IL-22 axis may have a protective role in the early stages of the SARS-CoV-2 infection, but may shift to a harmful response over time.^45^ Understanding the biology of IL-22 in lung health has led some authors to suggest using this interleukin as an immunotherapeutic strategy for COVID-19.^43^

Studies have shown that IL-22 can reduce the severity of pneumonia through immune regulation and tissue-protective or regenerative functions.^46^ ^,^ ^47^ As COVID-19 is a respiratory disorder with similar pathological features and symptoms comparable to other severe pulmonary virus infections, it is reasonable to speculate that IL-22 may also serve to limit the severity of this illness. Such findings further suggest that Tc22 cells expressed by IL-22 are greater in the age group of 0 to 12 years with asymptomatic disease course and uncomplicated adult cases, indicating that IL-22 has a protective effect.^47^ In contrast, the association between increased Tc17 cells and COVID-19 severity may reflect the negative impact of IL-17 and IL-22 coexpression.

Further research is needed to elucidate the specific mechanisms and functions of IL-22 in regulating the pulmonary microenvironment and its involvement in pro-inflammatory processes before it may be used as a new immunotherapeutic strategy for COVID-19. Higher levels of IL-22 expression in immunised individuals compared to those with moderate to severe COVID-19 suggests that this molecule may have a protective role in reducing disease severity in vaccinated individuals. These conclusions, however, are limited by the small sample size analysed; therefore, such findings must be explored in larger cohorts of vaccine recipients.

The WHO’s declaration that the COVID-19 health emergency has ended does not indicate the end of the pandemic. SARS-CoV-2 continues to circulate at alarming rates, contributing significantly to global mortality. Novel Variants of Concern (VOCs) continue to present a barrier to the development of effective vaccines. Therefore, understanding the immunological mechanisms underlying vaccination responses is crucial for designing immunotherapeutic interventions to control this global health crisis.
