# Supplementary material for: Assessment of immune responses to a Comirnaty® booster following CoronaVac® vaccination in healthcare workers
Source: Mem Inst Oswaldo Cruz. 2024 Sep 9;119:e230239. doi: 10.1590/0074-02760230239 (PMC11385826; doi:10.1590/0074-02760230239)
Supplement: Supplementary file 1 [file 1678-8060-mioc-119-e230239-s.pdf]

TABLE I

Adjusted mixed linear model to estimate the mean for fluorescence reduction neutralisation assay (FRNA) for each time point

Time	Estimated mean	95% confidence interval	
		Lower	Upper
0	81.7	54.1	123.2
1	18.2	11.8	27.8
2	3831.1	2550.5	5754.4

TABLE II

Mean fluorescence reduction neutralisation assay (FRNA) estimates: comparison between time points

Contrast	Ratio	95% confidence interval		p-value*
		Lower	Upper	
0/1	4.315	2.491	7.476	<0.001
0/2	0.022	0.012	0.037	<0.001
1/2	0.005	0.003	0.009	<0.001

*Tukey method for comparing a family of three estimates.

TABLE III

Spearman correlation analysis between fluorescence reduction neutralisation assay (FRNA) and IgG and IgA

Time	FRNA-IgG		FRNA-IgA	
	rho	95%CI*	rho	95%CI*
0	0.60	0.42; 0.73	0.44	0.21; 0.63
1	0.64	0.47; 0.77	0.41	0.17; 0.60
2	0.60	0.40; 0.76	0.48	0.27; 0.65

*Bonferroni-adjusted confidence intervals.

TABLE IV

Comparison of fluorescence reduction neutralisation assay (FRNA) distribution according to sex, presence of risk factors, and age

	Time					
	0		1		2	
Variables	Estimated difference	p-value*	Estimated difference	p-value*	Estimated difference	p-value*
Sex (Male-Female)	-3.28	1.000	2.47	0.741	976.00	0.173
Risk factor (No-Yes)	4.06	1.000	-4.20	1.000	-92.03	1.000
Age (<50- more or equal 50)	19.06	0.596	3.01	0.705	481.00	0.903

*Bonferroni-adjusted p-value.

CC BY

TABLE V

Median values of the 19 cytokines from coronavirus disease 2019 (COVID-19) patients and vaccinated healthcare workers (HCV)

	COVID-19 patients		Vaccinated HCW		
Cytokine	pg/mL Median (IQR)	n**	pg/mL Median (IQR)	n**	p-value
GM-CSF	48.50 (37.01-120.90)	17	60.24 (35.05-155.6)	14	0.8832
IL-2	33.40 (19.70-88.17)	14	46.82 (18.73-73.72)	9	0.8895
IFN-α	2.550 (1.212-7.994)	38	1.111 (0.7680-2.337)	6	0.0571
IL-4	21.44 (16.04-37.36)	17	27.73 (13.81-36.47)	7	0.9632
IFN-γ	31.38 (21.09-54.34)	78	23.83 (16.22-32.68)	24	**0.0331***
IL-5	21.45 (16.04-37.35)	21	41.19 (20.09-58.22)	10	0.1263
IL-6	77.16 (31.66-182.4)	75	176.7 (37.59-370.9)	20	0.1629
IL-9	21.28 (10.71-50.64)	40	34.20 (20.69-54.36)	16	0.1769
IL-10	7.508 (3.715-17.12)	92	7.034 (2.777-12.79)	34	0.5318
IL-17A	6.389 (4.211-13.57)	40	6.768 (3.427-10.20)	20	0.6206
IL-13	7.922 (5.470-16.57)	49	12.58 (6.452-26.19)	16	0.2343
IL-18	92.65 (57.64-172.8)	118	43.84 (28.78-86.70)	110	**<0.0001***
IL-21	33.52 (20.68-77.81)	41	31.25 (19.24-96.53)	24	0.9114
IL-22	55.19 (34.24-99.37)	25	95.06 (77.99-362.1)	13	**0.0122***
IL-23	23.85 (18.91-24.47)	22	35.99 (25.68-46.80)	4	0.1445
IL-1β	9.372 (4.297-15.35)	26	12.41 (6.310-33.59)	11	0.3780
IL-27	40.93 (31.22-83.19)	30	49.56 (26.10-113.9)	10	0.7759
MCP-1	187.7 (102.5-355.3)	120	116.6 (64.73-178.7)	117	**<0.0001***
TNF-α	18.59 (13.39-37.47)	58	23.99 (12.63-58.19)	18	0.4536

*$p < 0.05$; **n = number of samples with detectable amounts of cytokine. IQR: interquartile range; GM-CSF: granulocyte-macrophage colony-stimulating factor human; MCP-1: monocyte chemoattractant protein-1; TNF-α: tumour necrosis factor; IFN-α and IFN-γ: interferons and interleukins (IL-1β, IL-10, IL-13, IL-17A, IL-18, IL-2, IL-21, IL-22, IL-23, IL-27, IL-4, IL-5, IL-6, IL-9).

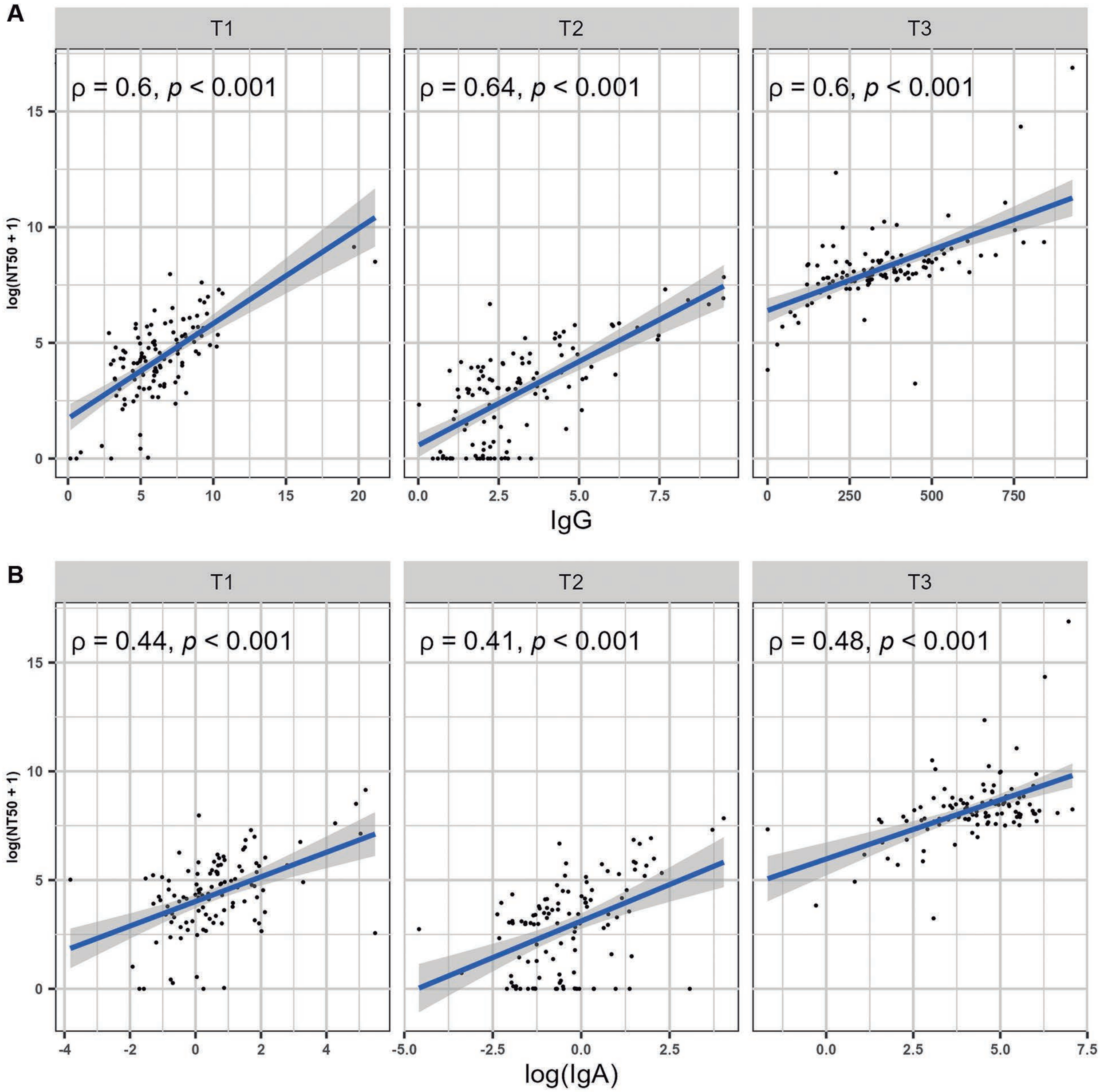

Fig. 1: spearman correlation IgG (A) and IgA log10 (B) index with NT50 (NT50 + 1) log10 values. Note: T1: 40 days post-primary vaccination; T2: 90 days post-primary vaccination; T3: 15 days post-booster vaccination; NT_{50}: neutralisation titres that inhibit 50% of severe acute respiratory syndrome coronavirus 2 (SARS-CoV-2) infection.

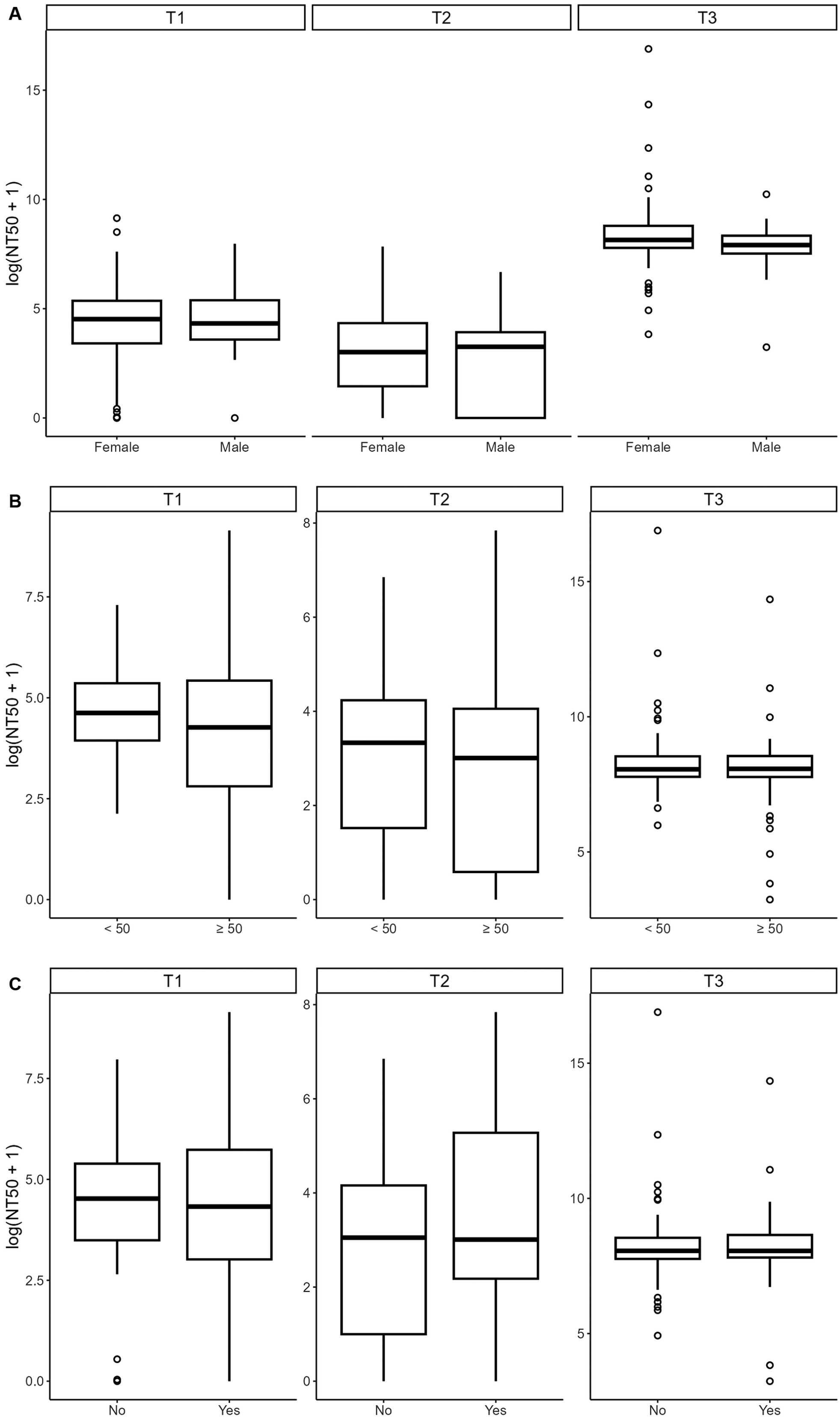

Fig. 2: comparison between the titers of 114 anti-severe acute respiratory syndrome coronavirus 2 (SARS-CoV-2) (NT_{50}) tests by Sex (A), Age (B), and Presence of risk factor (C) protein S antibodies at three different data collection times. Note: T1: 40 days post-primary vaccination; T2: 90 days post-primary vaccination; T3: 15 days post-booster vaccination; NT_{50}: neutralisation titres that inhibit 50% of SARS-CoV-2 infection.

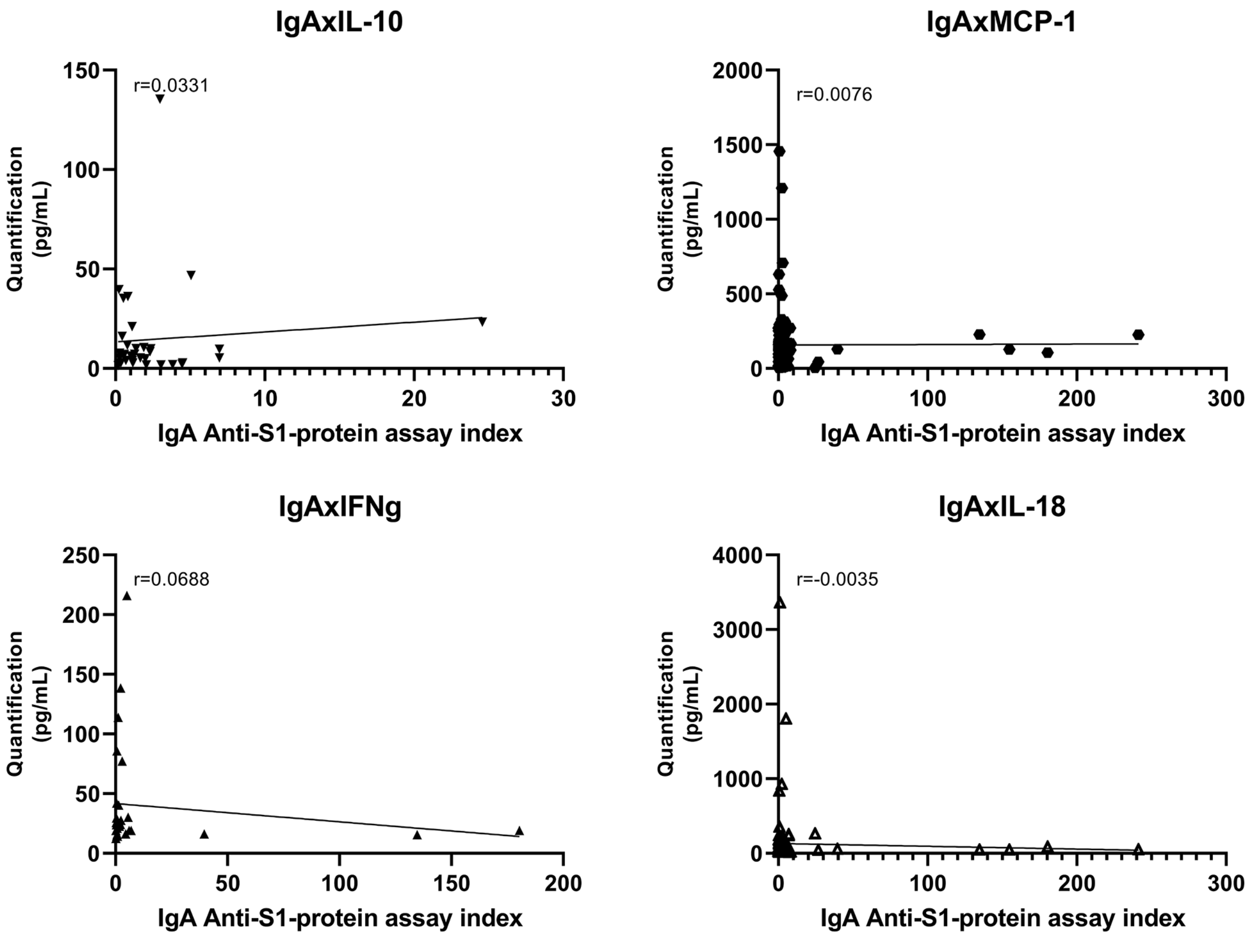

Fig. 3: spearman correlation graph between IgA anti-severe acute respiratory syndrome coronavirus 2 (SARS-CoV-2) and cytokine and chemokine dosages in vaccinated healthcare workers (HCWs). Note: IgA: immunoglobulin A; MCP-1: monocyte chemoattractant protein-1; IL: interleukin; IFNg: interferon-gamma.

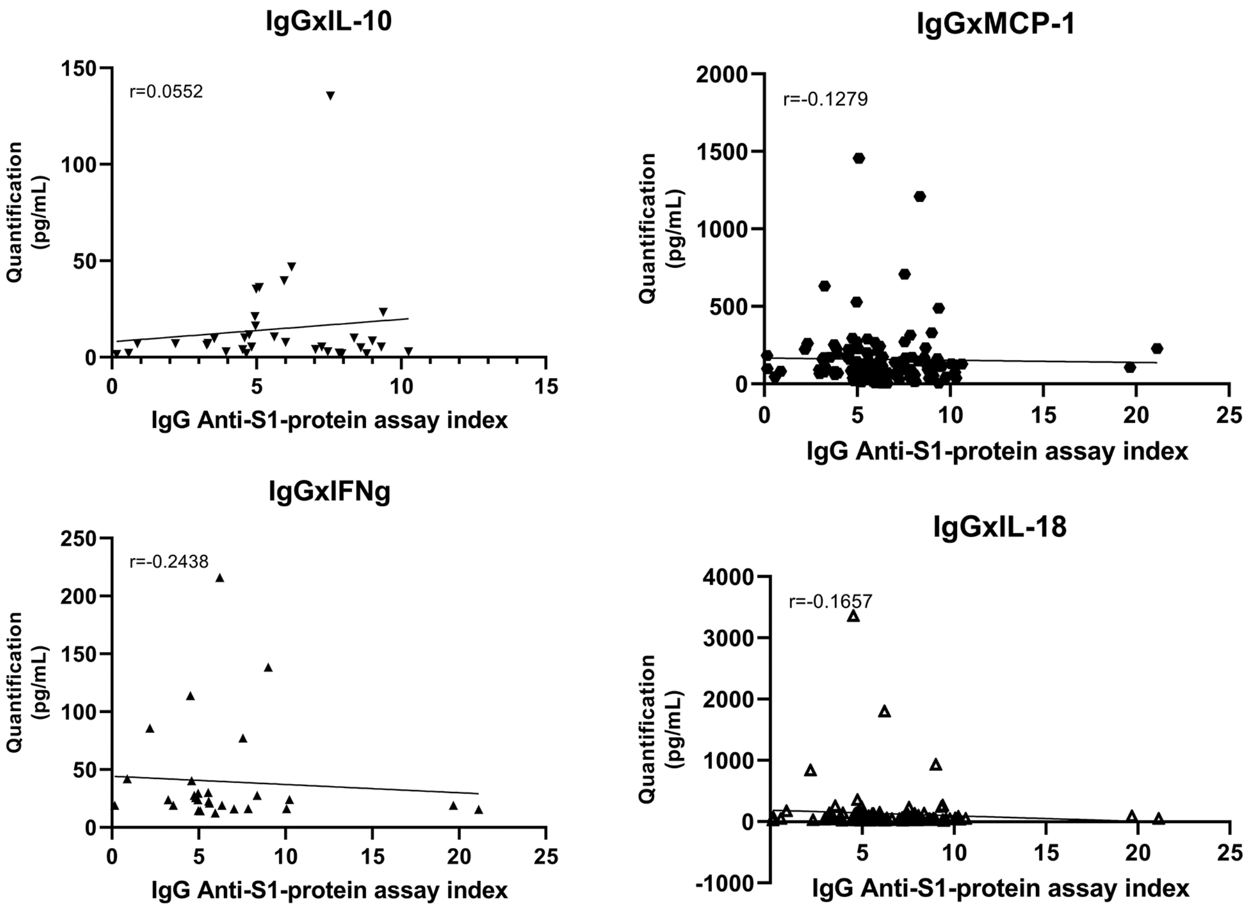

Fig. 4: spearman correlation graph between IgG anti-severe acute respiratory syndrome coronavirus 2 (SARS-CoV-2) and cytokine and chemokine dosages in vaccinated healthcare workers (HCWs). Note: IgG: immunoglobulin G; MCP-1: monocyte chemoattractant protein-1; IL: interleukin; IFNg: interferon-gamma.
